# Patients' representation of oncological disease: psychological aspects in the cancer journey

**DOI:** 10.3389/fpsyg.2023.1087083

**Published:** 2023-07-12

**Authors:** Ilaria Durosini, Gabriella Pravettoni

**Affiliations:** ^1^Department of Oncology and Hemato-Oncology, University of Milan, Milan, Italy; ^2^Applied Research Division for Cognitive and Psychological Science, IEO, European Institute of Oncology IRCCS, Milan, Italy

**Keywords:** cancer, psycho-oncology, social support, doctor-patient communication, cancer patients and survivors

## Introduction

Cancer patients' emotional distress, anxiety, and depression may affect different life areas (Rowland et al., 2009; Park et al., 2017; such as work, family, and relationship contexts) and may also lead to lower adherence to the intervention and detrimental effects on treatment efficacy (Arrieta et al., 2013; Savioni et al., 2022). In addition, people who received this diagnosis may change their motivation for everyday activities and their identity (Faccio et al., 2020; Durosini et al., 2021a). Some patients report having difficulty returning to their lives after cancer, experiencing the diagnosis as a trauma (e.g., Carreira et al., 2018). These consequences can occur regardless of the patients' stage of cancer and can also emerge during the communication of the diagnosis.

The confrontation with a life-threatening diagnosis requires physical and psychological adaptation to a new situation (Sebri et al., 2021; Durosini et al., 2022). In this sense, it becomes more and more important to understand not only the actual diagnosis from a medical point of view but also from the patients' personal and subjective representation of the disease. However, such subjective representation of the disease may lead patients to perceive themselves as “high-level” or “low-level” patients, with negative consequences for their healthcare journey.

In this opinion, we report highlights of patients' representation of cancer, with a specific focus on three psychological factors that could impact this representation. The available published evidence allows us to structure our opinion on the role of these aspects and the importance of the psychological perception of patients during the cancer journey. Finally, we conclude each section by providing details on the benefits of the psychologist's role in the oncological context to allow better consideration of all the discussed aspects.

## Patients' representation of cancer

When it comes to patients' perception, tumors can highly vary in terms of size, stage of advancement, and severity of the diagnosis. The literature shows that a patient's perception of his or her disease is relatively independent of the objective and “scientific” characteristics of the disease. Historical work in this area has been done by Leventhal et al. (1980, 1984) and Bishop (1991), leading to a conception of illness representation (or “schema”) associated with the idea of “prototype” from the cognitive psychology of categorization (Rosch, 1999). According to this theory, people form idealized models of symptoms and other attributes associated with different diseases. These models are compared with experienced symptoms and diagnosis information, leading to personal representations that influence coping, entry into, and use of medical treatment, as well as adherence and evaluation of treatment effects.

Broadly speaking, patients' representation should be considered as a mixture of the clinical/pathological information the patient is actually able to grasp, his or her own interoceptive experience of symptoms and discomfort, and the impact of health management on the overall quality of life (Weinman et al., 1996; Williams et al., 2023). People may have different mental images of their cancer and can use different colors, shapes, sizes, consistency, and texture to reflect what they thought their cancer looked and/or felt like (Harrow et al., 2008). Furthermore, chronic disease cannot be disentangled from the emotional burden that notably affects one's cognitive representations. For example, having a tumor deeply changes one's relationship with his or her own body (Sebri et al., 2021), when previously unnoticed, physical sensations are suddenly perceived as potentially threatening and felt in correspondence with strong negative arousal. In accordance with classical research on emotions (Frijda, 2009), it is not possible to predict one's emotional response based on objective characteristics of a stimulus: some patients may be psychologically disrupted or traumatized by some life events (e.g., Durosini et al., 2021d), while conversely, others may show extraordinary resilience when facing tragic news. Thus, published evidence highlighted that patients could have an individual perception of their cancer, judging the importance of their health, caring behaviors, and health-related concern in a specific way. According to Williams (1997), people's beliefs about their illness, self, and others could lead to possible dysfunctional illness behaviors. Illness representation includes, for example, the consequences and the causes of cancer and the controllability of the oncological journey (Williams, 1997). These aspects could be related to patients' personal experiences of illness, which generate inaccurate beliefs about the disease and could lead to inappropriate behaviors. Additionally, dysfunctional illness behaviors could be influenced by the beliefs that people have about themselves or others, in terms of personal vulnerability, negative evaluation of their self-competence, or lower beliefs in their ability (Williams, 1997). For example, the belief to be “defective” may lead patients who receive a life-threatening diagnosis to avoid complying with healthcare treatment, passively accepting their illness (Moorey and Geer, 1989). The interaction of all these aspects and other non-illness-related beliefs contributes to creating people's unique representation of their illness. On these bases, patients can perceive their cancer as more or less severe and their need for treatment as more or less urgent. As highlighted in a recent review, people who receive a low-risk oncological diagnosis may perceive their diagnosis and their overall health as less severe than people with advanced cancer (Dickey and Grayson, 2019). This could lead patients to perceive that their diagnosis has less weight and less value than that of other patients, promoting the view that they are less worthy of recognition. This can be linked not only to the type and severity of the cancer diagnosis but also to the type of cancer treatment prescribed and the related side effects. As Williams (1997) highlighted, the beliefs about the illness could incorporate the consequences/seriousness of the disease. In this line, patients who receive treatments that are associated with fewer adverse side effects may perceive themselves as less entitled to seek help when needed or receive attention and care, even if they need physical and psychological support. These patients' representation of themselves as less valuable than other patients may affect health management and the overall quality of life. It is possible that patients' subjective representations of the disease will be influenced by complex factors and will promote unexpected attitudes or behaviors that deserve to be taken into account to orient them in their decision-making and healthcare journey.

In addition to these aspects, published literature allows us to describe additional psychological factors that could impact the construction of personal representation on the cancer journey. In this opinion, we detailed three factors that may influence the process differently and allow for possible dysfunctional illness behaviors.

### Social representation of disease and treatment

Patients are influenced in their personal representations of their conditions by the social context and the opinion of others. For example, text mining research focused on the communication around chemotherapy on Twitter shows that the social discourse is mainly structured around the utility and the side effects of this treatment, with tweets coming from patients more emotionally than those from health organizations (Zhang et al., 2018). In virtue of its reputation in terms of public discourse and also media representations (e.g., in movies), which often emphasizes the disruptiveness of its side effects, chemotherapy may be perceived as the “main” treatment for cancer or the treatment that is employed for the more severe cases. Patients or survivors who did not undergo chemotherapy may feel intimidated and insecure when confronted with other patients who did. They may feel like their cancer was “certainly” less severe, and therefore, their current demand for care and their need for expression and active listening (within the social context) are less important to meet than others. These beliefs may lead patients to fail to recognize the relevance of their physical and emotional burden related to the illness, and, consequently, to passively accept their diagnosis (Moorey and Geer, 1989). This could have a relevant impact on their general wellbeing.

In this line, psychologists have a crucial role to explore patients' concerns and beliefs about their diagnosis and the impact of social representation of disease and treatment on their psychological representation. Exploring and managing all these aspects are relevant to taking care of negative emotions and distorted representations of clinical conditions that could lead to negative outcomes and dysfunctional behaviors.

### Social support and social recognition

Perceived social support during cancer may be an important precursor of personal growth and psychological wellbeing. Social support promotes patients' engagement in activities and individual motivation as it creates social bonds that encourage personal reflection on their needs and objectives (Novick et al., 2011; Cho et al., 2020; Durosini et al., 2021c). However, recent international studies identified important aspects related to the impact of cancer on informal caregivers and family members (Lambert et al., 2019; Sun et al., 2019), who can be involved in a more or less intense way in the cancer journey of their loved ones. In some cases, the social group may be a source of strain and distress instead of help to cancer patients. Reactions of family and friends to a diagnosis of cancer are influenced by several factors (Flanagan and Holmes, 2000). Excessive dread and fear may cause friends and family to display avoidance and withdrawal behaviors or over-solicitous and overprotective behaviors toward the loved ones (Norbeck et al., 1991). Therefore, caregivers' behaviors toward the patient could change deeply after the diagnosis of cancer. For example, the caregiver suddenly becomes over-caring and over-supportive in daily life. A patient who is used to being autonomous and independent inside the relationship may paradoxically feel overwhelmed and unworthy of the received attention. Additionally, the presence of the loved ones and the attention received can constantly remind the patient of the bad diagnosis: “I would just prefer to not think about my cancer while others' attention reminds me of it”. On the contrary, it could happen that caregivers show an attitude of underestimation of the illness or estrangement from their loved ones, avoiding paying attention to the diagnosis and treatment. This could often represent safe mind “escapism” to preserve themselves from unacceptable negative emotions but can be perceived negatively by patients. This could appear as a greater devaluation of their suffering and illness, and some patients could perceive themselves as “low-level patient”.

The psychologist could help caregivers to focus on their emotions, heuristics, and bias and obtain greater control of their inner world. Psychologists must ascertain the caregiver's preferred level of engagement in the decision-making process and the consultation and be aware of possible barriers/facilitators to family participation. The psychologist could help explore the solution for inclusion according to personal desire and help caregivers provide adequate support according to their loved one's preferences (Laidsaar-Powell et al., 2018).

### Poor communication

A central aspect of the process of care is the communication between healthcare professionals and patients. The discussion about the diagnosis and the health treatments could represent a stressful aspect for patients and a potential source of emotional/affective discomfort, if not adequately managed. The absence of an effective and supportive place where patients can comprehend the clinical information could lead to a lower understanding of the rationale of their illness and a lower ability to create realistic expectations about risks related to their diagnosis. Generally, a diagnosis of cancer is associated with the expectancies of some potentially unfavorable events, such as pain, death, and loss of function (Parker et al., 2001). Psychological distress, depression, and anxiety could also lead to additional difficulties in managing several challenges related to their health journey and the treatment decision-making processes. The inadequate management of emotions might lead patients to be worried, confused, and anxious, and, consequently, make decisions based only on their negative feelings, avoiding a rational evaluation of their situation. The presence of emotional processing guided by expert psychologists is essential to have well-informed patients who can be actively involved in the decisions concerning their cancer journey (Oliveri et al., 2020). This aspect is also important because a survey found that the majority of physicians do not have a consistent strategy or plan when they convey bad news to their patients (Baile et al., 2000).

Emphasizing a collaborative discussion and involving patients in the decision-making process could lead to an accurate evaluation of health conditions and the subsequent plan of care. This is true for all the types and stages of cancer. In addition, non-advanced cancer patients (e.g., stages I and II) can be exposed to the risk of poor communication. Doctors may focus only on “favorable” clinical aspects of these types of cancer compared with other types of serious cancers (i.e., less invasive therapies than advanced cancers), avoiding the importance of all the psychological aspects connected with this diagnosis. In a context of a non-collaborative relationship between doctors and patients, this information could promote an incongruence between their “clinical information” and “psychological perception of their illness”, leading them to a sense of “inferiority”. This could make patients feel not authorized in their demand for care and in their need for expression and active listening. Patients may also perceive themselves as “less needy and worthy of care” than other patients with a more serious diagnosis. The active role of a psychologist in a multidisciplinary team could help a collaborative discussion that considers patients' inner words (and the role of emotions in the decision-making process and their illness perception) and supports physicians during communications (Oliveri et al., 2020). Using emotional intelligence (Durosini and Pravettoni, 2021; Durosini et al., 2021b) to stay in contact with patients could guarantee better patient education and an empathic context.

## Conclusion

This article highlighted the relevance of patients' representations of cancer and the impact of social context and healthcare aspects on their perceptions of care. Published evidence highlighted that patients can represent their cancer as more or less severe and their need for treatment as more or less urgent. Patients may perceive that their diagnosis has less weight and less value than that of other patients, promoting the view that they are less worthy of recognition or value as a human being. We described three complex factors that influence the subjective representation of the illness: (i) the social representation of disease and treatment, (ii) the social support and social recognition, and (iii) the poor communication.

It is important to guide patients to recognize their health conditions and reorient their representations of illness into their own life story. Exploring their personal beliefs and needs in relation to their cancer journey allows them to have personal control over their illness, helping them to transfer their skills to several areas of life (Sebri et al., 2020). In this context, the active role of psychologists is relevant for taking care of patients' emotions, supporting physicians during communication, and guaranteeing patient and caregiver education and information (Oliveri et al., 2020). Additionally, patients can benefit from peer support (Durosini et al., 2021c). It is based on a non-hierarchical relationship, and the interaction between individuals with similar characteristics is generally beneficial. The relationship between patients with a similar history of cancer can help to establish a sense of normalcy in patients' life and acquire new competencies.

## Author contributions

ID conceptualized the ideas presented in the article and wrote the first draft of the manuscript. GP edited the manuscript and contributed with important intellectual content. All authors contributed to the refinement of the manuscript and approved the submitted version.
